# Facilitators and barriers in the humanization of childbirth practice in Japan

**DOI:** 10.1186/1471-2393-10-25

**Published:** 2010-05-27

**Authors:** Roxana Behruzi, Marie Hatem, William Fraser, Lise Goulet, Masako Ii, Chizuru Misago

**Affiliations:** 1 Université de Montréal, Faculty of Medicine, Department of Social and Preventive Medicine, Montreal, Canada; 2 Department of Social and Preventive Medicine, Montreal, Canada; 3 Université de Montréal, Faculty of Medicine, Department of Obstetrics and Gynecology, Montreal, Canada; 4 Université de Montréal, Faculty of Medicine, Department of Social and Preventive Medicine, Montreal, Canada; 5 Hitotsubashi University, Department of International and Public Policy, Tokyo, Japan; 6 Tsuda College, Department of International and Cultural Study, Tokyo, Japan

## Abstract

**Background:**

Humanizing birth means considering women's values, beliefs, and feelings and respecting their dignity and autonomy during the birthing process. Reducing over-medicalized childbirths, empowering women and the use of evidence-based maternity practice are strategies that promote humanized birth. Nevertheless, the territory of birth and its socio-cultural values and beliefs concerning child bearing can deeply affect birthing practices. The present study aims to explore the Japanese child birthing experience in different birth settings where the humanization of childbirth has been indentified among the priority goals of the institutions concerned, and also to explore the obstacles and facilitators encountered in the practice of humanized birth in those centres.

**Methods:**

A qualitative field research design was used in this study. Forty four individuals and nine institutions were recruited. Data was collected through observation, field notes, focus groups, informal and semi-structured interviews. A qualitative content analysis was performed.

**Results:**

All the settings had implemented strategies aimed at reducing caesarean sections, and keeping childbirth as natural as possible. The barriers and facilitators encountered in the practice of humanized birth were categorized into four main groups: rules and strategies, physical structure, contingency factors, and individual factors. The most important barriers identified in humanized birth care were the institutional rules and strategies that restricted the presence of a birth companion. The main facilitators were women's own cultural values and beliefs in a natural birth, and institutional strategies designed to prevent unnecessary medical interventions.

**Conclusions:**

The Japanese birthing institutions which have identified as part of their mission to instate humanized birth have, as a whole, been successful in improving care. However, barriers remain to achieving the ultimate goal. Importantly, the cultural values and beliefs of Japanese women regarding natural birth is an important factor promoting the humanization of childbirth in Japan.

## Background

Childbirth is regarded as one of the most important events in a women's life, and it can, in turn, affect the rest of their life, both physically, and emotionally [1].

During the past decades, giving birth has been increasingly medicalized procedures in most of countries [2,3]. Pregnancy and birth were conceptualized as pathological processes that require intensive monitoring by a physician. Medical interventions in childbirth such as use of electronic fetal monitoring (EFM), epidural analgesia, amniotomy, induced labour, episiotomy, and elective caesarean section deliveries increased especially in the North America in the last decade and continue to increase [4-6]. These procedures reinforce the perception of the mother's role as patient and can reduce her sense of control over her body [2,3].

Humanization of childbirth is a unique approach which has been implemented whose target is to make childbirth a positive and satisfying experience for both the women, and their family as a whole [7-9]. This strategy is used to empower women and their care providers by taking into consideration humanized values such as the women's emotional state, their values, beliefs, and sense of dignity and autonomy during childbirth. Humanized birth can be advocated by reducing over-medicalized childbirths, empowering women and implying evidence-based maternity practice [8]. The literature describes the specific characteristics of a humanized birth as follows: One which promotes the active participation of women regarding decision making, and other aspects of their own care, one which takes advantage of the expertise of both physicians and non-physicians, and allows them to work together as equals, and one which involves the use of evidence-based technology and medical intervention [8-11].

The territory of birth and its socio-cultural values and believes towards birth can deeply affect birth practice. This includes the cultural and religious values and beliefs toward birth practices present in different environments, and how these might, and often do, affect birthing practices [1]. The physical and social differences between the Japanese and American cultures, for example, have to some extent explain difference is 'a priori' ideas concerning birthing as a whole, and in particular the humanization of birthing practices [12].

The Japanese national politics of reproduction have attempted to influence birth rates according to national need while considering motherhood as the most important contribution a woman could make to Japanese society [13].

The movement to improve overall mother-child health in Japan started in 1936, when a 'Married Women's Voluntary Groups for Mother-Child Health and Welfare' established thanks to the help of a community organization named "Imperial Gift Foundation". It continued with issuing a 'Maternal and Child Health Handbook' by local government in Japan and creating various types of maternal and child health services. This handbook is a medical monitoring card that actually aims to promote the empowerment of women, since women possess their actual health record and participate as agents in their own care [14].

Three years after World War II, many laws and programs were announced to provide better maternal and child health services to the community. In 1950, however, a high infant mortality rate of 60.1 per 1,000 live births was a preoccupation for government [15]. Between 1949 and 1958, community organizations and local governments set up "Maternal and Child Health Centers" in rural areas, allowing for the first time safe deliveries under midwives' supervision. Until the 1950s and early 1960s, it was common for women in Japan to give birth at home attended by a midwife. However, as in most countries, birthing practices soon underwent a rapid change, and delivery at home was slowly replaced by hospital deliveries [7,12].

Subsequently, the routine medical management of pregnancy redefined motherhood to include the early stages of pregnancy [13]. In 1965, The "Maternal and Child Health Law" was enacted. This law encompassed women before they became pregnant, and included their health management in a comprehensive maternal and child health program. In 1968, the Ministry of Health and Welfare of Japan provided funding to local governments to promote a grassroots campaign which included a group of women volunteers acting under title of "Maternal and Child Health Promoters" [15].

In 1994, an "Angel Plan," and "Five-Year Project on Urgent Day-care Measures" were established with the aim of providing a worry-free childrearing environment by the agreement of the Ministers of Finance and Home Affairs. The "Healthy and Happy Family 21" program was announced in 2000 with four major initiatives. They included assuring safety and comfort during pregnancy and childbirth, maintaining and improving child health care and medical services standards; and promoting the healthy emotional development of children and reducing the anxiety related to childrearing [15].

Recently, the Government of Japan, through Japan International Cooperation Agency (JICA) and the Bureau of International Medical Centers in Japan (IMCJ), has been attempting to improve the quality of care in Japanese birthing centers by reducing caesarean section rates, as well as implementing the humanization of birth practice, not only in Japanese birth setting, but in other countries [16]. Most of the implemented projects focus on the humanization of childbirth with training based intervention activities such what we can find in Brazil [16,17]. Moreover, JICA provides a "Reformer Training Course" to train community leaders to promote humanization of birth and provide a unified system of care through labour, delivery and the post-delivery period. The JICA provides opportunities for friendly interaction between Japanese midwives and medical services personnel from the local maternity facilities. The Bureau of International Medical Centers of Japan facilitates training for medical personnel, especially obstetric nurses and midwives from developing countries, to undergo short or long term training in Japanese maternity clinics about "Humanizing Maternity Care" [16-18]. A number of studies are presently underway by JICA to improve maternal and child health on a global scale and scientifically demonstrate the validity of the Japanese approach, including the "humanizing of childbirth"[15].

Despite the existence in 2005 of more than 388 birthing houses in Japan, 98.8% births took place in hospital or private clinics, while 1.2% of all babies were born in maternity home and home [19]. In the hospital setting, power and authority is vested uniquely in obstetricians, and they are the only professionals with access to specialized obstetrics technology. Japanese obstetricians, however, have a rather limited involvement in uncomplicated births, and are usually less inclined to use medical intervention, as they consider birth a physiological, rather than a potentially pathological event [12].

Birthing practices are obviously different in Japan from those observed in the United States as they tends to be more natural and it includes avoidance of anesthesia [19], while the perinatal outcomes remain among the best in the world. With an infant mortality rate of about 2.7 per 1000 live births, Japan has been considered to be the best place to give birth in 2009 [20]. In 1990, the caesarean section rate was 11% in Japan, compared to 23.5% in the United States. Japan's caesarean section rate has in recent years risen exponentially from 11% to 21%, but even so, the increase has been less than in North America (27%), and up to 30% in the US alone [21].

Previous studies focusing on the childbirth experiences of Japanese women in the United States [14,22-24] have shown that the birthing experiences in these two countries can prove to be very different. There is no parallel research being conducted on the subject of humanized birthing practices in Japan alone. How do the Japanese experience the humanization of birth in the birth settings that have already aimed at providing such a care? And, what are the barriers and facilitators encountered whilst implementing such care in the Japanese institutions?

The objectives of this study are to explore the Japanese experience of childbirth practice in different birth settings where the humanization of birth has been implemented as an institutional goal, and also to explore the obstacles and facilitators encountered in such a practice.

## Methods

### Study design, setting, participants

A qualitative field research design was chosen to study the diverse facets and dimensions of the concept of humanized childcare, specifically the barriers and facilitators that pertain to it.

The nature of qualitative studies allows researchers to choose the participants, and setting, on an opportunistic basis [25,26]. In this study, the settings were chosen specifically, as they were supposed to have already implemented the humanized birth care approach, implying that their professionals were familiar with the concept.

This study took place in the setting of nine birthing centers, consisting of: two Level 4 highly specialized hospitals, three tertiary University-affiliated and/or private hospitals, two Level 2 private hospitals, one Level 1 private hospital, and birthing homes in the Tokyo, Okayama, Atsugi, Kamakura, Chiba, and Kanagawa prefectures in Japan.

Level 1 hospital is only equipped to handle normal, uncomplicated pregnancies and deliveries. Level 2 hospitals have supplementary equipment and professionals who are trained to provide care for patients with a minimum pregnancy risk-potential. Level 3 hospitals, on the other hand, have the necessary equipment and staff required to manage very complicated births, including those with a risk of serious illness or abnormality requiring intensive care for the mothers, or the newborns, before, during, and/or after delivery. Level 4 hospitals provide a wide-ranging array of critical care practices for the newborn, and offer a full range of specialty services. Levels 2, 3, and 4 hospitals also provide care for uncomplicated births [27].

The Japanese supervisor of the present study who was also the host researcher of the primary author provided a list of professionals from different disciplines, and with various levels of experience, at the beginning of the study. The professional participants were carefully and specifically chosen with the aim of obtaining a broad range of perspectives on the concept under study from different disciplines with various levels of experience [25,26].

Most of the professionals who participated in the study had already experienced, or worked on, a number of humanized birth projects. Potential participants were furthermore contacted by telephone, and given a formal invitation to participate in the study. Sampling continued until saturation occurred. The relevant sample of chosen professionals for participating in the interviews and focus groups consisted altogether of five obstetricians, one pediatrician, one administrative health care professor, one academic midwifery professor, twelve clinical nurse midwives, and five midwifery students at a 1^st ^degree Master level.

Moreover, during field visits, a total number of nineteen women in different birthing center units, such as the prenatal, labour, intensive care, and postpartum units, were also invited to participate in the study. The women were also chosen purposefully to obtain a maximum level of sample variation with regards to age, education, and delivery method.

### Data collection

Data was collected through observation, field notes, semi-structured open-ended in-depth interviews, and conversational interviews with participants, focus groups and the documentary data such as data from meetings, diaries, and photographs taken.

Combinations of individual interviews and focus groups enhanced the richness of our data. Data gathered through individual interviews and focus groups were used to illustrate the added-value. The combination of focus group and individual interview data also helped us in the conceptualization of the concept of humanized birth; and convergence of the central characteristics of the concept across focus groups and individual interviews, which enhanced trustworthiness of findings.

The approval of the original proposal of this study was taken from the Research Ethics Board of Université de Montréal, that is the first author's institution, and the letters authorizing access to the settings were obtained by host researchers before commencing the research. Women participants were clearly informed by the researcher that they could refuse to participate in the study, or withdraw at any time from it, without any prejudice on their normal care. Consent to conduct and tape-record the interviews was obtained from each individual professional and patient participant, and all participants were assured that all information would be treated confidentially and that their identity would only be known to the researcher.

The primary author of this study, a midwife and Canadian PhD candidate, undertook participant observations later used as data for about 8 to 10 hours per day, 4 days per week, for a period of approximately 2 months. Her midwifery profession facilitated her ease of entry into the different birth settings and unit areas. During this time, she had the opportunity to take part in meetings between midwives and mothers in the prenatal and postnatal stages of pregnancy, as well as attending some prenatal and postnatal classes dealing with childbirth, yoga, and aromatherapy. During this time, she also attended a range of different meetings and conferences in Level 3 hospitals that dealt with prenatal care in Japanese birth centers. A translator or one of the host Japanese researchers accompanied her during field visits.

To gain a more candid insight into the views of health professionals with regards to humanized birth care, the author also conducted many informal interviews, and asked the professionals many questions which shed light on some issues that strongly pertain to this study. She also undertook a total number of nine semi-structured, in-depth interviews with professionals, each lasting 30 to 90 minutes, and formed four individual focus groups with midwives during field visits ranging from June through to August of 2008. All the individual interviews were conducted in English. However, the presence of a Japanese translator or companion, consisting of one of the two Japanese host researchers, amplified the trust and mutual understanding between the researcher and interviewee during the interviews. For the focus groups, a Japanese translator accompanied the investigator to actively translate from English to Japanese, and vice versa.

The questions addressed to the professional interviewees were as follows: How do you experience childbirth in your institution and what are the potential obstacles and facilitators experienced towards the humanization of childbirth practice in you institute?

In addition to professional semi-structured individual interviews, a total of 13 semi-structured interviews, each lasting 20-30 minutes, and one focus group with 6 women, lasting approximately 90 minutes, were conducted with pregnant women during the field visits. The numbers of interviewed women according to unites, was as follows: prenatal unit (1), labor (2), postpartum (14), and intensive care units (2). A Japanese translator was present during these to facilitate communication.

The main question addressed to the women in these interviews was: "Could you please tell me about your experience during your pregnancy and/or delivery?" Have you ever heard about humanization of birth? If yes, could you please describe to me how you feel about humanized birth? What is the meaning of humanized birth for you? To achieve a deeper understanding of how women experience childbirth, the researcher also sometimes intervened to ask clarifying questions, such as: "Could you tell me more about your feelings on the matter? Could you give me an example?

### Data Analysis

All the interviews were audio taped and transcribed carefully by the primary author. Then, all the transcripts and field notes were entered into a software package (Atlas.ti 5), which is designed to handle qualitative data.

Considering the aims and research questions, the primary author undertook an inductive content analysis approach to find out how do the Japanese experience the humanization of birth and what are the barriers and facilitators for such care in the settings that have already aimed at providing humanized care. In inductive analysis, the themes are strongly linked to the data themselves [28]. She focused on exploring the barriers and facilitators, while she immersed herself in the interview transcripts and let the categories emerge from the data. She carefully examined the data, and proceeded to categorizing the relevant themes and key issues which arose from it [29]. A theme shows something significant about the data in relation to the research question. In our study, the themes were rules, regulations and strategies, physical structure, contingency factors, and individual factors. She validated the themes or categories in the early process on some samples of text. She examined the clarity and consistency of the themes by the assessment of intra-coder agreements. To achieve intracoder reliability, she coded-recoded five random sample of interview transcripts within a 6 weeks interval and used the specific formula to obtain an intracoder reliability coefficient. The intracoder reliability coefficient was shown to be more than '0.90'. The main author consistently held discussions with the host researchers, referred back to the audio tapes and rechecked each category many times. She used a constant comparative method to make differences between categories clearly, means she did a systematic comparison of each text assigned to a specific theme or category with those assigned to that category before.

The primary author coded the data without trying to fit it into a pre-existing coding framework, or her analytic preconceptions. She proceeded from inferring a generalized idea of the participant's answers, to analyzing the data and finding codes. After this analysis, she proceeded on to reorder the simplified codes, or patterns found in the research, into novel subthemes which highlighted the essential qualitative experiences of the interviewees with regards to barriers and facilitators in humanized birth care. The subthemes were small units of the themes, for example, the subthemes of the theme *Rules and Strategies *and barriers were; *companion restriction *and as facilitators were; *preventing unnecessary medical interventions, natural methods of relieving pain, obtaining the women's consent, longer hospitalization time. *The subcategories were checked for reliability by Japanese investigators other than the primary author who did the coding.

The use of different data sources in the study, with regards to setting and time frame allowed the researcher to generate a more comprehensive analysis of the emerging data. The researcher did not have the possibility to do a member check or participant's validity, but she used a triangulation method of validating the findings. The findings from the different sources of methods such as in-depth interviews, informal interviews, focus groups, field notes and observations, and document draw the similar conclusion. To ensure that the data analysis in the study was systematic, the main author held consultation with the host researchers and was very meticulous while coding and analyzing the data, and achieved a consensus with host researchers on the analytic conclusions of the study. The primary author's background as a midwife, and her previous knowledge of the subject of humanized birth, represented a facilitator in helping her to accurately describe her reflections and conclusions on the research carried out.

## Results

The data collection was conducted in a total number of nine settings, and involved forty-four recruited participants. The mean age of the professional participants was 36.4, and ranged from 22 to 60 year-olds. Most of the midwives (12 out of 18), held a Bachelor's degree in midwifery. Five midwives were studying midwifery at the MSc, level and one had a PhD in midwifery. The mean years of clinical experience of the professional participants was 11.9 years, but this ranged from training status to up to 40 years of experience (Table 1).

**Table1:** Socio-demographic characteristic of professional participants

Characteristics	N [25]
**Age**	
	
Minimum	22
Maximum	60
Mean	36.4
	
**Education**	
	
Bach -Midwifery	12
MSc- Midwifery	5
	
Obstetric& Gynaecologist	5
	
PhD- Public	1
	
Health	1
MD-Paediatrician	
	
PhD Midwifery professor	1
	
**Experience**	
	
Minimum	0
Maximum	40
Mean	11.96

The mean age of the interviewed women was 29.1, and ranging from 17 to 41 years old. Most of them (16 out of 19) were housewives. Most women (14 out of 19) were also primigravida, and only 2 of 19 had a history of abortion. Twelve of the nineteen women interviewed went on to have a vaginal delivery, whereas 4 had a caesarean section, and only 3 were in the prenatal period (Table 2).

**Table 2:** Socio-demographic characteristic of Japanese woman participants

Characteristics	N [19]
**Number of pregnancies**	
	
First	14
Second	3
Third	1
Fourth	1
	
**Age**	
	
Minimum	17
Maximum	41
Mean	29,1
	
**Education**	
	
Primary	1
Secondary	9
University/college	9
	
**History of abortion**	
	17
Non	1
one	1
two	
	
**Marital status**	
	
Married	19
Single	0
	
**Job**	
	
Yes	3
No	16
	
**Mode of Delivery**	
	
Vaginal	12
Caesarean section	4
no delivery	3
	
**Reason for Caesarean**	
	
NA	15
FHR abnormality	1
Hypertension	1
Myoma	1
Placenta abnormally	1

The characteristics and information related to the childbirth practices in the different studied Japanese birthing units where humanized care has been instated are shown in Table 3.

**Table 3:** The specific characteristics of the setting regarding to childbirth practice

Characteristic Presence of characteristic: + Absence of characteristic:-	4^th^level Hospital (1)	4^th ^level Hospital (2)	3^nd ^level Hospital (1)	3^nd ^level Hospital (2)	3th Level Hospital (3)	2^nd ^level Hospital (1)	2^nd ^level Hospital (2)	2^nd ^level Hospital (2)	Birthing home
									
Average childbirth per year	800	1500	500	550	500	1100	1200	1200	120
Transferring rate to hospital	NA	NA	NA	NA	NA	NA	NA	NA	3-4%
**Medical Interventions**									
									
C-section rate	27%	30-35%	< 15%	< 15%	40%	< 15%	< 15%	< 15%	NA
Epidural analgesia	-	40-50%	rare	rare	< 10%	-	-	-	-
Routine episiotomy for first pregnancy	-	-	-	-	70%	-	-	-	-
Continues Electronic Foetal Monitoring (EFM)	high risk	high risk	-	-	high-risk	-	-	-	-
Routine IV line in normal pregnancy	-	-	-	-	-	-	-	-	-
									
**Physical Environment**									
									
LDR room	-	+	+	+	+	+	+	+	+
Tatami style room	-	-	+	+	+	+	+	+	+
Joining room for family	+	+	+	+	+	+	+	+	+
Private Post partum rooms	+	+	+	+	+	+	+	+	+
Shared postpartum rooms	+	+	+	+	+	+	+	+	-
Common labour	+	-	-	-	-	-	-	-	-
									
**Main care Providers**									
									
Nurse-Midwife	+	+	+	+	+	+	+	+	+
Obstetric& Gynaecologic	+	+	+	+	+	+	+	+	-
Nurse	-	-	-	-	-	-	-	-	-
									
**Continuity of care**									
									
Care is provided by the same care provider during prenatal	-	-	-	-	-	-	-	-	+
									
**Natural methods of relieving pain**									
									
Birth pool	-	-	+	-	-	+	+	+	+
Aromatherapy	+	+	+	+	+	+	+	+	+
Massage	+	+	+	+	+	+	+	+	+
Moxa therapy	-	-	-	-	-	-	-	-	+
Emotional support by companion	+	+	+	+	+	+	+	+	+
Emotional support by midwife companion	+	+	+	+	+	+	+	+	+
Changing position and saddle seat	+	+	+	+	+	+	+	+	+
Listen to music	+	+	+	+	+	+	+	+	+
Breathing techniques	+	+	+	+	+	+	+	+	+
									
**Rules/Regulation**									
									
Eating during labour	+	+	+	+	+	+	+	+	+
Conditional companion during labour and delivery	+	+	+	+	+	+	+	+	+
Companion in C-section	-	-	-	-	-	-	-	-	NA
Cutting umbilical cord by father	-	-	-	-	-	-	+	+	+
Having kid's companion	+	-	-	-	-	+	+	+	+
Companion in post -partum period	-	-	-	-	-	-	+	+	+
Staying baby in mother's room in first 24 h after delivery	+	-	+	+	+	+	+	+	+
Breastfeeding within first hours of life	+	+	+	+	+	+	+	+	+
Rooming-in	+	+	+	+	+	+	+	+	+
Average duration of hospitalization after C-section	9 days	9 days	9	9 days	9 days	9 days	4 days	4 days	NA
Average of hospitalization after normal vaginal delivery	6 days	6 days	6	6 days	5 days	7 days	2 days	2 days	5 days
Free style position in delivery	-	-	+	+	+	+	+	+	+

With regards to medical intervention practices in the studied settings, only the Level 4, and one of the Level 3 hospitals, the ones which also acted as referral centers for high risk pregnancy cases, had caesarean section rates over 25% ( range: 27-40%), whereas the other centers had rates less than 15%. Only two of the Level 4, and two of the Level 3 hospitals provided epidural analgesia for pain relief, but only in specific cases, or following a woman's demand for it. The ratio for these two circumstances was 40-50%, and 10% respectively. Other hospitals were seen to rarely, or never, use this method of pain-relief.

None of the centers were practiced routine episiotomies, except for one of the Level 3 hospitals, which provided routine episiotomies for 70% of primipara cases, and none of the centers used continued EFM or routine intravenous infusion in normal pregnancies.

Regarding the physical environment, or setting, only one of the Level 4 hospitals, and the birthing clinic, had no Labour Delivery Recovery room (LDR), and with the exception of the Level 4 hospitals, the centres usually included Tatami-style (traditional type of Japanese flooring) rooms. All the centres had both private and common postpartum rooms, with some exceptions, where the postpartum rooms were only of the private kind.

The main maternity care providers present in the hospitals and clinics were the nurses/midwives, and obstetricians/gynaecologists. None of the centers provided continuous care either, except for the birthing home. With regards to pain relief, all of the nine centres readily provided natural methods of relieving pain such as birth pools (4 of 9), and other comfort measures methods, such as aromatherapy, massages, position changing, saddle seats, emotional support by companions and midwives, music therapy, and breathing techniques (9 of 9).

The rules and regulations regarding childbirth practices in these centres, allowed women, to eat during labour, unless there was a contraindication, as in the case of a planned caesarean section birth. The hospitals also permitted women to have a companion during normal deliveries, provided that they had attended the prenatal classes.

However, seven out of the eight hospitals, did not allow any companions, not even the husbands, to attend the caesarean section deliveries. As well as this restriction, only three out of the nine centres studied allowed women to have a companion during postpartum, and four out of nine did not permit children in the LDR or postpartum rooms.

Except for the Level 4 hospitals, all the centers also authorized women to choose a free-style position during delivery, and three out of the nine centers allowed the fathers to cut the umbilical cord at birth, even though this practice is officially illegal in Japan. All the centres had rooming in, and breastfeeding was initiated during the first 24 hours after delivery. One of the Level 4 hospitals, however, was an exception to this and they separated the mother from the baby for the entire first day after delivery. The mean recorded average time of hospitalization was 5.3 days for a normal uncomplicated pregnancy, and 8.1 days for a caesarean section.

### Definition of humanized birth

From the professionals' point of view, humanized birth is not perceived as a restriction in using medical intervention, but it involves all aspects of care that provide a good physical and psychological status for the patient:

"Humanized birth is not a case without any medical intervention. Sometimes we need medication [...] we should marry humanized birth with medical intervention just by explanation, communication and the maintaining confidence". (P13-M3: Midwife in focus group)

Most of the academic interviewees recognized that humanized birth cannot possibly be limited to a specific definition, nor can it be seen as a long list of tasks that need to be performed. Referring to the statements below, made by an obstetrician (P6) and a midwife (P3) respectively, humanized birth provides continuous improvement in the way health professionals promote care as one human being interacting with another human being:

"We think that humanization of childbirth is a process, is a transition for each of woman, professional, person, family. We do not define humanized care as this or that specific action or approach. Humanization of birth affects mother and family. After birth, it continues. It is different, depend on culture and place". (P6-Obstetrician)

"For me there is no specific definition, always it changes. It is important to listen to the mother's voice, to listen to the family's voice, what is the best for the woman and the family". (P3-Midwife)

However, women interviewees believed that the humanized birth means respect to the mother's decision and desires:

"It means one's own will, women's own will. If I want to have baby in this way, others should respect me... I feel childbirth is my own affair, not others'. I will deliver; I will have a baby by myself. If people around me accept my will... I can rely on them. It is important to give attention to what I want to do? " (P1-w1-Woman)

### Barriers and Facilitators

Analysis of the data collected in this research revealed some of the barriers and facilitators encountered by the humanized birth practice. These aspects can be categorized into four main groups: Rules, Regulations and Strategies, Physical Structure, Contingency Factors, and Individual Factors.

Table 4 is a description of the overall analysis summaries on barriers and facilitators of humanized birth practice in the studied settings not a comparison between them. The presence or absence of each critera was identified through an inductive content analysis of the interview transcripts, the filed notes as well as the documents collected during field visits.

**Table 4:** Barriers and Facilitators in Humanized Childbirth

Characteristics Presence of characteristic: + Absence of characteristic:-	1st level hospital	2nd level hospitals	3th level hospitals	4th level hospitals	Birthing home
**1. Rule and Regulation**					
- Prevention from having a companion in postpartum	-	-	+	+	-
- Prevention from having a companion in operation room	-	+	+	+	NA
- Banning of children from the mother's rooms	+	+	+	+/-	-
- The restriction in the number of companions allowed during labor or delivery	+	+	+	+	-
- Conditional acceptance of the father as a companion	+	+	+	+	+
					
**2. Physical Structure**					
- Common Labour and delivery room	+	-	-	+/-	-
- Common postpartum room	+	+	+	+	-
					
**3. Contingence Factor**					
	+	+	+	+	NA
- University-affiliated hospitals					
- The lack of midwifery authority	+	+	+	+	+
	+	+	+	+	+
- Mal practice litigation					
	+	+	+	+	NA
- Physicians' training and medical skill	+	+	+	+	-
- Overcharge of work for care providers	+	+	+	+	-
- The workplace demand on Japanese men	General condition	General condition	General condition	General condition	General condition
					
**4. Individual Factors**					
- Lack of decision-making by women in hospitals	+	+	+	+	-

**Facilitators in Humanized birth**					

**1. Rules and Regulation**					
- Preventing unnecessary medical intervention					
- Natural methods for relieving pain	+	+	+	+	+
- Getting the women's consent	+	+	+	+	+
- Long stay in birth setting	+	+	+	+	+
	+	+	+	+	+
					
**2. Physical Structure**					
- LDR room and other facilities	-	+	+	+/-	+
					
**3. Contingence Factor**					
- Midwifery system	+	+	+	+	+
- Private and public health care system	+	+	+	+	+
					
**4. Individual Factors**					
- Women's culture, values and beliefs in natural birth	+	+	+	+	+

### 1. Rules and Strategies

Some rules and strategies which have been categorized as barriers for the humanization of birth practice correspond to the rules present regarding companion restrictions. The facilitators, on the other hand, in this group of factors, include the prevention of unnecessary medical intervention, natural pain-relief methods used in most of the settings, rules regarding women's consent, and longer post-partum hospitalization times.

#### Barriers

##### • Companion restriction

*Prevention from having a companion during hospitalization *in the Mother-Foetus Intensive Care Units (MFICU) and postpartum, as well as the *banning children *from the mother's rooms, were the most important barriers showed in the 3^rd ^and 4^th ^Level hospital settings. As a whole, postpartum women showed a great preference for a longer amount of time spent with their chosen companions. Surprisingly enough, the majority of Japanese women, seem to be used to the state of this situation, however. The following statement was made by a multiparous woman who had been hospitalized during her first pregnancy in the MFICU in a Level ^3 ^hospital because of an amniotic fluid leakage accident:

"Sometimes I would like to see my husband and my 3-year old daughter, but the kids are not permitted in this hospital. I can understand, however, that a hospital is not a good place for kids, especially when their mother is in this state". (P1-Woman)

*The restriction in the number of companions allowed during labour or delivery *was regarded as another barrier in this setting. A head midwife in a Level 3 hospital pointed out that:

"Most specialized hospitals don't allow the mother to have her kids, parents, or friends as their companion" [...] "Parents can assist in labor, but during delivery, only the husband can assist. We are thinking about opening up the possibility of having the presence of parents during delivery; however, this is an issue that worries us because of the possibility of chaos in the hospital environment." (P1- M1: Midwife)

*Prevention from having a companion in the operating room *in all the studied settings except for the Level 1 hospital was another strong barrier encountered in this group of factors, and seemed to act as a significant stress factor for the women, while they prepared for the caesarean section operation. Quote:

"The mother had some anxiety about the anesthesia and pain, but I reassured her that she would not feel any pain. She became happy when she heard this. The mother went to the caesarean room on foot. The mother's family was there, but they did not accompany the mother to the operation room, as it was banned for even the husband to accompany his wife". (P1: field note in a 3^rd ^level hospital)

*The conditional acceptance of the father as a companion during delivery *in hospitals and birthing homes, given that they have attended prenatal courses, is also considered a huge barrier as most of these classes are held during working hours, and thus most of the fathers are not available to attend them.

#### Facilitators

##### • Preventing unnecessary medical intervention

Preventing unnecessary medical intervention, such as the use of routine EFM, epidural analgesia, intravenous infusion, etc. was the main strategy used to implement humanized birth care in all the studied settings. All the obstetricians interviewed concurred with the policy of limiting the administration of epidural injections unless there arose a specific need to control anxiety and/or high blood pressure in the patient. A paediatrician in a Level 4 hospital had this to say on the matter:

"This hospital provides more natural births. Many women choose this hospital for natural births. Even foreign women, when they come here, they see how natural birth is important. We believe that only some women need epidurals, for example, anxious women...". (P1-Pediatrician)

In an informal interview, a midwife in a Level 3 hospital stated:

"In this hospital, they check the fetal heart rate for about 45-60 minutes, and if everything is ok, there is no more need for monitoring". (P1-Midwife)

##### • Natural methods for relieving pain

Almost all the settings studied here provided natural methods for relieving pain. These included: massages, breathing techniques, thermo therapy, birth pools, aromatherapy, warm blankets, and emotional and psychological support from companions. A midwifery professor who was training midwifery students in a Level 3 hospital mentioned:

"We provide a comfortable environment and give massages, baths, put pressure on vital points on the woman's body, warm up the mother's feet, and help her relax. We believe that foot baths can promote the mother's contractions". (P7-Midwifery professor)

##### • Getting the women's consent

Acquiring the women's consent before assisting in their labour or delivery was mandatory for all the care providers and students involved in the hospitals and birthing homes. Midwives and obstetricians stated that this rule was aimed at respecting the women's privacy and dignity. One of the obstetricians in a Level 2 hospital said:

"We must keep patient privacy and patient-family privacy. Of course, in the hospital, in the past, we have had many people attending labor or delivery, for example: midwives, nurses, doctors, interns, residents, the staff surrounding the patient, etc., and there was no privacy. Now, we ask the patient if the presence of staff or students is allowed or not. This way, pregnant women can concentrate on the delivery and her family, and a nice delivery environment is provided". (P5-Obstetrician)

##### • Longer hospitalization time

Longer stays at the hospital and birthing homes (9-14 days after caesarean section and 5-7 days after normal pregnancy) were mainly perceived as strong facilitating strategies for humanized birth care, as it provides women with a comfort time which they can use to recuperate, and to get used to their new lives. Midwives also stated that during this period, they carry out daily breast massages in the centers to facilitate breastfeeding. Quote:

"She must stay at the hospital for at least two weeks, as in Japan, there are many small families with parents mostly in another city, leaving mothers alone and helpless, and thus making the staying time at the hospital long. Mothers say that after three days of caesarean section, they can just now go to the toilet". (P1: field note in a 2^nd ^level hospital)

### 2. Physical structure

The most prominent physical structure factor that can be perceived as a barrier in this study is the use of a common labour, delivery, and postpartum room. The presence of an LDR room, however, is seen as a facilitator.

#### Barriers

##### • Common Labour and delivery room

A common labour and delivery room, as seen in one of the Level 4 hospitals, nominated as a baby-friendly hospital, and at one of the Level 1 hospitals, where the delivery room was actually shared between two or three women, is considered a barrier for humanized birth care. Because of this obvious barrier, women and their husbands/companions had no privacy whatsoever during labour, and women had only a limited space for walking and changing position. Quote:

"The environment was friendly but not a spacious place. There weren't any LDRs, and one labour room was shared between three mothers. One of them had given birth 30 minutes ago and the baby was in the Kangaroo position on the mother's chest, one of them was expected to be in full dilation, and one of them was in the early stages of labour". (P1: field note in the 1^st ^level hospital)

##### • Common postpartum rooms

Common postpartum rooms that were shared between 4-6 women, and the lack of privacy therein, were seen as further barriers regarding humanized birth in the hospitals. Women were restricted from bringing their companions outside of visiting hours, since there was just not enough space, even if the beds were separated by a curtain.

#### Facilitators

##### • LDR rooms and other physical factors

Most of the settings studied provided a physically and emotionally-friendly environment for women by providing access to fully-equipped LDR rooms, and various other facilities. The LDR rooms were generally extremely large and spacious, and equipped with a TV, a refrigerator, and a private bathroom. Some of them also had Tatami-style mats, and/or birth pools. One of the Level 2 hospitals had Tatami-style LDR suites equipped with kitchens. The traditional Japanese method of dealing with pain, which involves grapping a cord as if hanging from it, was also provided in one of the Level 2 hospitals, and the birthing home. Interestingly, one of the Level 4 hospitals also had the initiative, and the available facilities, to allow and encourage family members to stay with the mother in the mother-infant intensive care units. This was a service that was not available in the other hospitals. A midwife in that particular Level 4 hospital stated that:

"We encourage family, specially the husband, to come to the hospital and spend his time with the mother. We provide a bed for the father or kids if they want to stay at night. Sometimes we call the husband at work and ask him to come into the hospital". (P1-M2: Midwife)

All the settings studied also chose a joyful pink or green colour for the birthing unit, and they made use of spacious, well-equipped adjoining hospital rooms to provide a relaxing and pleasant environment for the women and their families to meet during the appointed visiting hours.

### 3. Contingency factors

In this category, relevant barriers include: university-affiliated hospitals, lack of midwifery authority in the birthing centers, malpractice litigation, physicians' training, and workplace demands. The midwifery system, and the presence of both private, and public health care systems, is considered contingent facilitators in the scope of this study.

#### Barriers

##### • University-affiliated hospitals

University-affiliated hospitals were seen as a potentially important barrier for humanized birth programs, as their regulations interfere with continuous care. Only the birthing home provided such care, which was carried out by the midwives of the center. Midwife participants in one of the Level 1 hospitals complained about the interruption of care because of student trainees in the birthing units.

"This hospital accepts trainees, and we cannot stay with mothers all the time and sometimes the mother has not the same midwife or the same obstetrician during continued care [...] The Midwife who admits the woman may not be the same midwife who will attend her delivery". (P9- Midwife)

##### • The lack of midwife authority in hospitals

The lack of midwife authority in Japan can, in some cases, prevent midwives from accurately and efficiently accomplishing their required tasks, even during normal pregnancies. The autonomy of midwives tends to be quite limited in hospitals in Japan. While midwives are responsible for normal pregnancy and delivery, no intervention procedures are allowed to be carried out by them, not even episiotomies, or second degree laceration repair. A midwife from a Level 2 hospital involved in one of the focus groups stated:

"In high risk pregnancies, obstetricians (or doctors) are powerful and don't allow midwives or nurses to do anything [...] in Japan, midwives cannot prescribe medication" (P12-Midwife)

"Midwives are responsible for normal pregnancy and delivery. No intervention or even IV infusion can be prescribed by midwives". (P1: field note in birthing home)

##### • Malpractice litigation

The increase in the number of lawsuits was the main barriers in achieving better humanized birth care observed by the interviewed obstetricians. One obstetrician in a Level 2 hospital mentioned the fact that the fear of lawsuits brought about a bias in the general decision-making process of the obstetricians, who consequently are more inclined to use unnecessary medical intervention methods to prevent unwanted outcomes. Quote:

"Recently, barriers are legal issues. All staff have many responsibilities toward the safety of the pregnant women and their family, and sometimes decision making is based on legal issues, not humanized based. Recently, suing has been increased... doctors are afraid of being sued". (P5- Obstetrician)

##### • Physicians' training

The approach to training and educating physicians is yet another potential barrier for humanized birth care as classically-trained physicians are taught to use all their medical skills during birth. Interviewed obstetricians stated that the Japanese health-care system has actively invested in reducing caesarean section rates, by training expert obstetrics physicians not to use this method, even in difficult cases. However, one of the obstetricians in a Level 1 hospital also brought forward the argument that it would take a long time to change the current medical education system and thus the doctors' behaviour on this matter, to implement a better humanization of childbirth. Quote:

"In the process of becoming obstetricians & gynecologists, we trained with the concepts of using technology and proper medicine. A doctor should use the proper medical instruments and medication to become a doctor. This is the way that medical professors and staff train students to become obstetricians. I also was in such an environment for many years. When I got out of such a medical center, at first I never thought of humanized birth. I thought, that is a very different, a very dangerous way of delivery, it is not a good way. During my work at tertiary hospitals, I never had a chance to even think about what was happening in birth after discharge from the hospital, we just talked between professionals and doctors, not patients".(P6- Obstetrician)

##### • Workplace demands

Workplace demands on care providers were considered as a barrier for providing further humanized care in the hospital settings. Midwives in the Level 3 hospitals generally concurred that the overcharge of work, and the number of tasks to be carried out by them in the hospitals, were the main reasons for the lack of continuous professional support for women during their hospitalization:

"There is one mother in the room. They have a lot of time, but are alone in their rooms. During the day we are in the nursing station. We cannot stay with the mother in this part. We are pretty busy [...] we cannot provide continuous care. We have a lot of work to do and just don't have time. We need to check up on three or four pregnant women at the same time". (P8- Midwife)

One of the women interviewed in a delivery room in a Level 3 hospital, this being her first pregnancy at the age of forty-one, had this to say on the matter:

"I would like it if the midwives were here all the time. I was admitted three hours ago and the midwives have been visiting me every 20-30 minutes and giving me advice on how to breathe, however, when I came here, the midwife stayed in my room for one hour". (P1-W7: Woman)

The obstetricians in the hospitals also generally complained about the overwhelming amount of work, and the lack of time allotted to establishing good communication grounds and a good personal relationship with the women. Quote:

"I used to visit 30 patients in an hour that means 2-3 minutes for each. If we visit even 5-6 women in one hour, it is not so good for the hospital. They ask us to visit at least 6 to 10 patients in an hour". (P6- Obstetrician)

The workplace demands on Japanese men also acts to prevent fathers from accompanying their wives during delivery, another barrier encountered in this group of factors. A 28 year-old mother, giving birth to her first baby, stated that her husband was able to accompany her only for a few hours after delivery, but could not stay the night because of a demanding work schedule. However, she seemed to find this a positive note, since she didn't want him to miss work, and thought this would give her more time to rest at night.

#### Facilitators

##### • Midwifery system

The presence of midwives as the main care providers in normal pregnancies in Japan is a facilitating factor for humanized birth in all of the childbirth settings studied. While the nurses are generally busy undertaking various administrative tasks in the hospitals, obstetricians are free to fully engage in dealing with high risk pregnancy cases. In Japan, women are allowed to choose between midwives and obstetricians as the professional that will be most involved in their delivery. In general, women seem to prefer the midwife as their main care provider as they feel that they have a more practical approach to childbirth, even in cases where caesarean sections are necessary. Quote:

"After the caesarean section, the baby was shown to the mother and the midwife touched the mother's hand with the baby's lips. The mother felt as if the baby had kissed her hand. The baby was then moved to another room and the family came to see it. The midwives changed the music to birthday music, and encouraged the father to hug the baby and take photos. A few minutes later, the operating staff gathered around the mother and sent a greeting and appreciation to mother for the baby's birth. Mother was encouraged to show her feelings." (P1-field note in a 3^rd ^level hospital)

##### • Private and public health care systems

After analysing the midwife interviews, and the observatory field notes, we were able to conclude that the mixture of both private and public healthcare facilities in Japan, and the rather extensive variety of birth settings available to women there, acts as a definite facilitator in the implementation of humanized birth care. The healthcare costs between the private and public settings in Japan are almost on a par with each other, as the public healthcare system is not free of charge there. Nevertheless, this choice allows women to have more options at their disposal when it comes to delivering their baby, with regards to both setting, and preferred healthcare provider, which is indeed a clear facilitator for the further implementation of humanized care. Quote:

"In Japan, the mother is the one who chooses where she gives birth. Women can call hospitals and ask to visit before deciding to pick them. They may visit many hospitals and consult many people before deciding on one". (P1- field note) "Another facilitator is the fact that women can not only choose where to give birth, but with whom as well". (P11-M1: Midwife in a focus group in a 3^rd ^level hospital)

### 4. Individual factors

The main barrier found in this group was the lack of decision making by the women, while culture, values and beliefs were categorized as an individual facilitator factor.

#### Barriers

##### • Lack of decision-making by women in hospitals

Women's lack of full participation in the decision-making process, and their generally passive role in the hospital setting, is considered an individual barrier when dealing with humanized birth. One of the obstetricians stated that the women were keen to obey orders because of a religious belief in the Buddhist notion that 'there exists an inter-relationship between people and nature'. The interviewed academic professor, however, disagreed with the speculation of a Buddhist ideological influence on the women's decision-making process:

"I think it is the Japanese attitude and culture (not Buddhist ideology) that brings Japanese people to accept other's decisions and rarely disagree. When you compare Japanese people with others, you see that the Japanese are more likely to obey decisions made by others. Patients also have difficulty talking to doctors, they are afraid to talk to them, they usually just say 'thank you very much', even when they have a problem or a question". (P2- Administrative health care professor)

Professionals seemed to agree that women should by all means highly trust their physicians, but should also be informed of any relevant proceedings and be allowed to participate in decision making. They also agreed that the women should be provided with enough information to give them the ability to fully understand their own diagnosis and treatment, and be vigilant of the risks and benefits of each procedure, as well as being able to choose between the different delivery options. The physicians also often emphasized the fact that pregnant women should have the choice to be able to decide the outcomes by themselves, but in reality they do not. In Japan, in this case, the woman's decision still strongly depends on the physician's or midwives' ultimate decision.

#### Facilitators

##### • Women's culture, values, and beliefs regarding childbirth

The women's culture, values, and beliefs regarding childbirth, were considered facilitating factors for humanized birth in Japan. All the Japanese women participants seemed to agree with the belief that 'a pregnant woman is not a sick person, and thus childbirth is not a sickness', and natural birth, specifically the minimal use of analgesia in the hospitals, was a highly valued factor in this circle. Quote:

"Women in Japan culturally avoid having a lot of medical intervention. It is traditional to believe that during the time that the baby is in the tummy, we should avoid medication". (P3- Midwife)

Interviewed midwives stated on this matter that generally Japanese women preferred not to get rid of their pain completely, but rather often opted for pain relief through natural methods.

"In contrast to American women, Japanese women believe that pain is necessary for delivery. (P7- Midwifery professor)

"In Japan, we know the childbirth experience is very important, and we should remember it after delivery. In Japan, labour pain is not bad." (P14-M1: Midwifery student in a focus group)

Finally, one of the obstetricians also added:

"In Japan, pain has a Buddhist meaning. The act of dealing with the delivery pain is a meaningful act, and overcoming pain is seen as important. A great Buddhist said that pain should be controlled by the mind. Pain during labor is important. Labor is described as 'JINTSU' and it means pain in the battle room." (P1-O1: Obstetrician)

## Discussion

The present study details some of the characteristics of the current Japanese birth setting, and the barriers and facilitators that birthing centers which have implemented a strategy for humanized care in this country, have encountered. Considering the results of the study, all the care provided around the time of birth that promote the physical and psychological health of women and respect their desires and needs, can be defined as humanized care.

Our results have so far shown that the humanization of birth care in Japan is greatly supported by innate cultural values regarding childbirth, as well as other beliefs, such as the strong drive seen in most of the participating centers, to provide a completely natural birth, and to prevent unnecessary medical intervention in the case of uncomplicated births. Given all these positives, however, we must take into account that some barriers are still in play concerning the further development of this strategy. Barriers include factors such as companion restriction in the birthing units, amongst others.

The birthing home was found to be a unique place that fulfilled all of the requirements of the description pertaining to humanized birth. These requirements include: continuous emotional and psychological care and support during pregnancy and postpartum, the avoidance of unnecessary medical intervention methods, and the empowering of women by allowing them to actively participate in the decision making regarding their own experience [7]. Our results support the Matsuoka's demonstration of characteristics of birthing homes in Japan means the importance of waiting for the baby to be born and valuing the labor pain. On the other hand, waiting means respect to a woman's physiological process of birth and avoid any interventional strategies such as induction of labour or episiotomy. Furthermore, waiting for labour allows women to give birth with their own strength and confidence in their bodies [19].

Our results showed that in the settings studied, the use of electronic foetal monitoring (EFM) was restricted to high risk pregnancy cases, and that only the Level 4 hospitals and one of the Level 3 hospitals, the ones that also acted as referral centers for high risk and very complicated pregnancies, had a high level of caesarean section operations. A study previously carried out by Fiedler had also noted that methods of medical intervention such as EFM, epidural analgesic use, induced labour, episiotomy, and instrumental deliveries, are relatively common procedures in abnormal or 'difficult' births in Japan, compared to a very low level of intervention for normal births [12]. The reduced utilization of the technological interventions in normal pregnancies could be explained by the fact that, in Japan, both women and obstetricians consider birth as being primarily physiological rather than potentially pathological [22]. On the other hand, unlike their Western counterparts, Japanese obstetricians and women are generally reluctant to use the medical interventions or modern diagnostic technologies that are available in most of hospitals [13]. According to previous research, Japanese obstetricians' attitudes towards birth tend to advocate humanized birth as they take into consideration the social and psychological conditions in which the women spend their pregnancies. Japanese obstetricians strongly emphasize the women's responsibility during pregnancy. Often women receive much advice from midwives and obstetricians to lower levels of physical and mental stress in their lives and maintain a more relaxed lifestyle to prevent future problems [13].

Our results show the use of an electronic monitoring (EFM) restricted in high risk pregnancies, and only the fourth level hospitals that were referral centers for high risk and very complicated cases, had a high level of caesarean section. Fiedler's study showed the medical assistance and interventions such as EFM and epidural analgesics induced labour, episiotomy, and instrumental deliveries are common procedures in every abnormal or 'difficult' birth in Japan [12].

Apart from the Level 4 hospitals studied, all of the settings respected women's autonomy and provided them with the ability to choose a desired birthing position, and/or to have a free-style labour and delivery. The women were also provided with the choice between either Tatami style, or ordinary gynaecological beds for delivery. The possibility of choosing a free-style position during labour and delivery, acted as a boost for women's confidence, and provided them with a sense of control, and the feeling of having played an active role in the birth of their child [30].

Japanese women's beliefs toward natural childbirth, and the impact of their cultural views on this matter, also acted as a facilitator for the implementation of humanized birth in Japanese hospitals. The way pain is perceived is different across cultures. In Japan, labour pain is considered as an important and necessary element of childbirth as a woman grows into motherhood through experience of pain [19]. Most women are automatically expected to choose and undergo non-pharmacological methods of pain relief when it comes to childbirth, and are usually readily prepared to control and endure their labour pains through the use of natural methods such as breathing, listening to music, remaining emotionally calm, soaking in a hot bath, and requiring aromatherapy [14]. Women believe that they confronting labour pain using natural approaches to control pain is the best approach [19]. Watanabe's study demonstrated that the rate of analgesic delivery was around 2.1% in Japan in 2005 [31]. Ito's study demonstrated that Japanese women, even when giving birth in America, usually preferred to turn down analgesics, compared to American women, which were keener on receiving epidural injections for pain relief. Interestingly, the participating Japanese women in Ito's study became so confused when they were offered a choice of painless delivery as they thought that a natural birth is the best [14]. Sahrts Engel, in her article, depicted her experience of pregnancy and childbirth in Japan as follows: "it is wildly accepted that the mother's stoic endurance of pain enhances valuing and bonding with the baby and appreciation of one's mother. Also, endurance upholds the family"[22]. The importance of providing one-on-one care and support to woman by a midwife has an important role in reducing labour pain in Japanese women [19].

In Japan, the average hospital stay after a vaginal delivery is about one week [24]. Our results support this allotted time frame as a positive factor in the implementation of humanized care. This long postpartum stay is valued throughout the Japanese healthcare system. In Ito's study, it was also shown that Japanese women who gave birth in the United States were generally unsatisfied with the American system of early discharge, and stated that they felt the need for a longer recovery period after delivery [14]. The hospitalization of women for the required length of time after delivery is widely considered as one of the basic principles of maternity care, as this postpartum time allows the healthcare providers to fully and effectively monitor the mother and baby completely, and to provide the mother with the essential information and assistance necessary during the first days of motherhood [30]. Hech et al. showed, that women who left the hospital earlier than the recommended time, were more likely to terminate breastfeeding altogether, than women who stayed the full length of time (relative risk: 1.11, 95% confidence interval 1.01, 1.23). The authors of this article speculated that this disparity could be because of the fact that mothers leaving the hospital setting earlier did not receive adequate assistance with regards to breastfeeding techniques [32].

Midwives were seen to be the main care providers in normal pregnancy cases in all the studied settings. With regards to this matter, it is of interest to note that the Japanese childbirth system is more similar to the Western European and England systems [12]. The role of midwives in the Japanese and European systems is replaced by the role of nurses in the American maternity care system [10,33]. We have concluded from this study that the presence of a qualified midwife in the birth setting plays an essential part in humanized birth care as a whole. In Japan, despite the midwives' implemented right to be able to practice their profession autonomously, the reality and exercising of this right, is far from ideal [10,19]. The lack of authority that midwives possess in hospitals, and the restrictions imposed on common midwifery tasks such as the prescription of routine medications, the ability to perform episiotomies, or repairing lacerations, is concluded in this study to act as a large barrier for the implementation of humanized birth care both in birthing homes, and in hospital settings.

The lack of full participation in the decision-making strategies by Japanese women in the birth settings were also found to act as a barrier to humanized birth care in the scope of this study. This aspect of humanized care was seen to act as a factor which disempowered the women with regards their own labor and delivery. Furthermore, this factor could consequently lead to an increase in staff responsibilities, which can snowball toward a rise in physician authority, and in turn even less autonomy for the women involved. Page states that: "When women are involved in making decisions about their care and they have a good relationship with staff, they are more likely to feel confident in their abilities to mother the baby" [10].

A previous study by Ito also showed that "Japanese women tend to trust their treatment decisions to physicians, and seem to generally be more obedient than American women"[14]. Fiedler also noted that women in the hospitals seem to take a more 'passive role' after being admitted to the delivery room [12].

The largest barrier encountered in the implementation of humanized birth care in the course of this study was found to be the lack of companionship during hospitalization in the hospital and birthing home settings. The conditional permission system instated with regards to the husband's or family's ability to attend the delivery acted as a significant impediment to humanized birthing care. Humanized childbirth emphasizes a need for access to a continuous pool emotional and physical support during the pregnancy, labor, and postpartum stages. The importance of a companion is even more prominent when the hospitalization periods are so long for women, as is the case in Japan. The presence of family members during child birth experiences has been recommended by WHO as one of the main aspects of humanized care [33]. The benefits of continuous one-on-one support by a companion during labor have also been noted by a Cochrane systematic review [34], and other research reviews in the past [35,36]. The presence of a companion or family members in the ICU or operating rooms has also been shown to provide positive psychological support for the pregnant women, whilst the care providers concentrate on the task of attending to more medical issues during the procedures.

Further researchers have argued this point. Hodnett, for example showed in one of his studies that women who had a companion who was not a member of the hospital staff present during labour and delivery, were less likely to require analgesia, and were generally more satisfied with their childbirth experience [34]. Leslie and Storton, in another research, also showed that the unrestricted presence of a birth companion of the women's choice, including husbands, partners, children, family members, and friends, was a key component regarding women's satisfaction during their birth experience [36]. In spite of the relevant research results, in Japan, husbands are still not always permitted to attend births, although in most centers, they will bypass this rule with the condition that they participate in the prenatal classes [37]. Japanese fathers, however, are usually very busy, and workplace demands often impede their participation in all the required prenatal classes, especially the ones held during working hours. Because of this impediment, the fathers are usually not given the opportunity to attend their own child's birth [14].

In conclusion, the results of this study aim to shed light on the facilitators and barriers to the humanization of birth in Japanese institutions. Further research is still required in this field of study, however, to study and compare results with centers that have already promoted a higher level of healthcare quality, but which haven't yet set humanized care as their ultimate goal.

The results will help to clarify the potential barriers and opportunities for improving the humanization of child birth practice in the studied setting in Japan. Although the results of this study are not intended to be generalized, Japanese institutions and healthcare providers, as well as administrators and decision makers, can certainly benefit from the results found herein, and use this knowledge as a tool to improve childcare practices in all birth settings, regardless of their degree of specialty. Despite the fact that in the present study the humanization of childbirth practice and its facilitators and barriers have been limited to Japanese birth settings, some of the problems discussed in this article, might still be relevant to obstetricians, midwives, nurses, and a range of other maternity care providers in other countries. Care providers, specifically nurses and midwives, should attempt to fully respond to both physical and psychological needs of the women.

## Limitations

This study had several limitations:

First, as the sample size in this study was relatively small, the interviewed professionals and birth settings cannot be taken as representative of all healthcare professionals, or birth settings in Japan. However, representativeness is not often the primary objective of a qualitative study.

The lack of direct observation of actual deliveries in the chosen birth settings, also acted as another limitation. In the settings studied, even when there were deliveries occurring at the appropriate time of the field visits, the women refused the presence of strangers in the delivery rooms.

In our study, the participants' difficulty in expressing themselves in English was one of the biggest restraints experienced during the data collection period. Even in the presence of a Japanese translator, or the host researcher, which made us more confident about the mutual exchange of information between the interviewer and the interviewee, there arose some communication problems as there were some words or phrases in Japanese, and in English, that had no accurate equivalent translation. The Japanese "honorifics" that are used as suffixes attached to the ends of words (-*sama, -dono, -san, -kun, -chan) *has no English equivalent. Honorifics are a manner of showing your feelings in relation to others and depending on how they are used; they can be either respectful or offensive. Our Japanese translator was very cautious in interpreting these honorifics to English, however, there is always a risk when undertaking this type of research, to lose some data or important information because of a lack of equivalent in the languages.

We believe, however, that our research has managed to minimize this risk by introducing two Japanese supervisors and/or host researchers into the study, to avoid the effects that the What North American culture might bear on the interpretation of data and results. I would finally like to note that previous literature have also supported the findings in this study.

## Conclusions

Based on our results, we concluded that despite the increased rate of caesarean sections in the highly specialized hospitals studied, the participating Japanese settings for which humanized birth care has been an institutional goal, have proven very successful in providing this type of care in term of preventing unnecessary medical interventions. Our results allow us to conclude that the cultural values, beliefs and views of both participant women and obstetricians towards birth is a strong facilitating factor for the humanization of the childbirth practice in this setting. While in most of the developed countries, enduring a painful birth is considered an outdated idea in the presence of analgesic drugs, labour pain is still not seen as useless in Japan, but a physiologic process that produces something invaluable to the mother and baby.

Even so, some barriers remain to achieving a more humanized form of childbirth in Japan, Future challenges, especially in the birthing homes could be the implementation of the strategies that provide more autonomy to midwives, including an extension of the scope of midwifery practice, such as give injections or medications with permission of a physician or obstetrician in the cases that a normal birth takes a sudden turn to a risky situation. Moreover, all the strategies to diminish the pressure on husbands in the work place and allow them to accompany their spouse during prenatal, intrapartum and post partum could be helpful in providing more psychological and emotional support for women. Meanwhile, changing birth settings rules and regulation in order to accept the presence of husband in labour without any precondition is necessary for providing more humanized care.

## Footnotes

The primary author was awarded by Japan Society for Promotion of Science (JSPS) and Canadian Institute of Health Research (CIHR) to accomplish this research, in summer 2008.

## Competing interests

The authors declare that they have no competing interests.

## Authors' contributions

Six persons have fulfilled the conditions required for authorship. RB has coordinated the paper from writing its protocol, taking approvals, designing the semi-structured questionnaires, collecting the data, transcriptions, analysis, and redaction of the manuscript. MH helped in qualitative analysis and validate the methodology, and participated in drafting the manuscript. WF participated in drafting the manuscript. LG participated in the design of the study and questionnaire development. MI managed the coordination of the surveys. CM was supervisor of this research and helped in preparing the fields of research, helped in data collection, and participated in drafting the manuscript.

## Pre-publication history

The pre-publication history for this paper can be accessed here:

http://www.biomedcentral.com/1471-2393/10/25/prepub
